# Phenotypic Modulation of Adipose-Derived Stem Cells and Fibroblasts Treated with Povidone–Iodine and Chlorhexidine in Mono and Coculture Models

**DOI:** 10.3390/biomedicines11071855

**Published:** 2023-06-29

**Authors:** Alina Chelmuș-Burlacu, Eric Tang, Dragoș Pieptu

**Affiliations:** 1 Plastic Surgery Department, “Grigore T. Popa” University of Medicine and Pharmacy, 700115 Iași, Romania; dragos.pieptu@umfiasi.ro; 2 Phoenix Biomedical Ltd., Macclesfield SK10 3HZ, UK; erictang1307@gmail.com; 3 Plastic Surgery Department, Regional Oncology Institute, 700483 Iași, Romania

**Keywords:** povidone–iodine, chlorhexidine, adipose-derived stem cells, fibroblasts, coculture

## Abstract

Topical antiseptics are essential in wound treatment, and adipose-derived stem cells (ADSCs) have recently been proven to facilitate healing. However, the impact of antiseptics on ADSCs has not been fully elucidated, especially in relation to other relevant cell types present in the wound microenvironment, e.g., fibroblasts. This study evaluated the effects of chlorhexidine and povidone–iodine on four cellular constructs in 2D and 3D in vitro culture systems. Cell constructs were treated with two concentrations of each antiseptic, after which cell migration activity, α-SMA, and Ki67 marker expressions were assessed and compared. Both tested concentrations of povidone–iodine impaired migration and sprouting compared to chlorhexidine, which had minimal effects when used in low concentrations. The gap in the wound healing assay did not close after 24 h of povidone–iodine treatment, although, at the lower concentration, cells started to migrate in a single-cell movement pattern. Similarly, in 3D culture systems, sprouting with reduced spike formation was observed at high povidone–iodine concentrations. Both antiseptics modulated α-SMA and Ki67 marker expressions at 5 days following treatment. Although both antiseptics had cytotoxic effects dependent on drug concentration and cell type, povidone–iodine contributed more substantially to the healing process than chlorhexidine, acting especially on fibroblasts.

## 1. Introduction

Wound treatment involves antiseptic usage both for cleansing in acute settings and for chronic management [1,2]. Guidelines recommend antiseptics to lighten the bioburden in infected or critically colonized wounds, as well as prophylactically in the case of high-risk wounds [2,3,4,5]. Typically, practitioners choose which antiseptics to resort to based on their experience, local guidelines, and economic factors. Currently, the most frequently used antiseptics in wound management are octenidine, polyhexanide, iodophors, sodium hypochlorite, taurolidine, and nano-silver [1,2]. Chlorhexidine is another option that is particularly useful for cleansing and preventing the colonization of burn wounds [6,7]. Overall, the available literature suggests that the impact of antiseptics on the wound healing process is complex and nuanced (e.g., reports of cytotoxicity in vitro and heterogeneous results from in vivo and clinical studies) [3,4,6,8,9].

Povidone–iodine (PVP-I) is a broad-spectrum antiseptic that releases iodine in aqueous solutions, disrupting the normal function of the bacterial cell membrane and mitochondrial respiratory chain through protein denaturation [4,10,11]. It is the first choice of antiseptic in case of bites, stabs/punctures, and gunshot wounds [1,2]. In chronic wound management, the indication of PVP-I is under discussion. Although some authors recommend it for chronic wounds with critical colonization or biofilm, others find it unsuitable for such purposes, mainly due to its cytotoxicity [1,2,12]. To exert a meaningful bactericidal effect, the minimal contact time needed is 10 s, and efficacy is maintained down to a concentration of 0.001% [3,5]. Although PVP-I presents multiple advantages over other antiseptics, some studies suggest that it may actually have a negative impact on the wound healing process. Thomas et al. demonstrated that PVP-I impairs fibroblast migration, proliferation, and metalloproteinase expression [3]. Liu et al. evaluated migration patterns for different cell types following PVP-I treatment. At 1% concentration, the gap in an artificial wound assay remained open, even after 14 days, compared with the control samples of untreated cells, where the gap closed after 24 h [13].

Chlorhexidine (CHX) is a biguanide antiseptic with broad-spectrum efficacy that does not include *Pseudomonas aeruginosa* and *Proteus mirabilis* [14,15,16]. CHX works by displacing divalent cations from membrane phospholipids, thus altering membrane fluidity and permeability, with potassium efflux [17,18]. It is used mostly in burn wound management, but not all practitioners prefer it [7]. In a survey conducted by Abdel-Sayed et al., 54% of healthcare professionals, mainly from Europe and the Middle East, claimed to use CHX on burn wounds, but also alternatives, such as Betadine^®^ in which PVP-I is the active substance (40%), saline solution (11%), soap and water (6%), and hydrogen peroxide (3%) [6]. The recommended contact time for bactericidal effect ranges from several seconds to 2 min, and the concentration can vary from 1% to 0.002% [3,16,18]. Although it is considered less cytotoxic, it seems that this antiseptic can stimulate inflammation and cell necrosis, delaying granulation tissue formation and healing, as with PVP-I [14].

Wound healing is a complex and dynamic process with three tightly regulated, overlapping phases: inflammation, proliferation, and remodeling [19,20]. Dysregulations lead to impaired healing that manifests clinically as chronic wounds and pathological scarring. One major factor that can disrupt the wound healing process is the bioburden within the wound microenvironment, and topical agents such as antiseptics can contribute crucially to wound management [12,20,21]. Although already widely used in clinical practice, emerging scientific data point to the potential inhibitory effects of antiseptics on wound healing, a hypothesis still being explored and debated [3]. Additionally, the research regarding the impact of antiseptics on scarring is currently limited but emerging reports suggest that CHX plays a role not only in how a wound closes but also in subsequent scar tissue formation. One study reported that CHX increased the expression of α-smooth muscle actin (α-SMA), a myofibroblastic marker, and down-regulated the Ki67 marker. The study concluded that CHX, apart from affecting cell proliferation, also seems to induce a pro-scaring response [22]. While Ki67 is a well-known proliferation marker, the α-SMA marker is highly expressed by myofibroblasts, cells associated with pathological scaring [22,23]. Thus, the extent to which antiseptics could impact the healing and scarring process is not fully known.

More recently, therapies based on stem cells have been attracting scientific and clinical interest once stem cells were found to exert pro-regenerative effects on wounds. Various stem cell types and delivery methods are being studied, including with a view to enhancing chemokine-mediated homing [24]. For instance, Fathke et al. reported that, following skin injury, 15–20% of dermal fibroblast originated from bone marrow progenitors that homed within the wound microenvironment [25]. The use of adipose-derived stem cells (ADSCs) is another emerging strategy in wound healing management, based on the observed ability of ADSCs to stimulate granulation tissue formation, increase capillary density, and promote the re-epithelization process [26]. The available data are limited regarding the impact of antiseptics on ADSCs, calling for caution and further research. In one such study by Kim et al., PVP-I was found to have a cytotoxic effect and, as such, to significantly reduce cell viability [27]. The precise effects on ADSCs within a wound microenvironment when antiseptics are used represent another insufficiently researched aspect.

Given the state of the art hereby summarized, the aim of this study was to evaluate the effects of two widely used antiseptics, PVP-I and CHX, on ADSCs and normal human dermal fibroblasts (NHDFs). This was carried out in 2D and 3D monocytic and coculture in vitro systems. Two coculture conditions with different cell ratios were set up to mimic real wound microenvironment scenarios. Cellular migration, sprouting, proliferation (Ki67), and α-SMA markers were assessed on four different cellular constructs.

## 2. Materials and Methods

### 2.1. Cell Lines

The cell lines used were normal human dermal fibroblasts (NHDFs, C-12302 Promocell, Heidelberg, Germany) and human adipose-derived stem cells (ADSCs, PT-5006, Lonza, Basel, Switzerland), grown in specific cell culture media, FGM-2 Bullet Kit (CC-3132, Lonza, Basel, Switzerland) and ADSC^TM^ Growth Medium (PT-4505, Lonza, Basel, Switzerland), respectively. For cocultures, a 1:1 mixture of the two media was used. The two cell types were transfected with different fluorescent protein markers using 3rd generation lentivirus vehicles. The lentivirus contained an elongation factor 1α as promoter, a puromycin resistance gene, and either a green fluorescent protein gene (LVP426, Amsbio, Abingdon, UK) for ADSCs or a red fluorescent protein gene (LVP429, Amsbio, Abingdon, UK) for NHDFs. The subsequently expressed green or red fluorescent protein was used to enable cellular tracking in the cocultures (data and protocol not shown herein). For all experiments, four ratios of ADSCs/NHDFs were used in the culture systems: monocultures (exclusively ADSCs and exclusively NHDFs) and two cocultures at 4:1 and at 1:4 NHDF to ADSC ratios (A20N80 and A80N20, respectively), as illustrated in Table 1.

To the current authors’ knowledge, there is no available report regarding the ratio between dermal fibroblasts and ADSC during wound healing. Thus, a ratio of 4:1 NHDFs to ADSCs was chosen based on Fathke et al.’s report [25]. The 1:4 NHDF to ADSC ratio was chosen in an attempt to mimic a scenario where ADSC would be employed from exogenous sources.

### 2.2. Antiseptics

Two of the most common antiseptics were chosen for evaluation in both 2D and 3D culture systems: povidone–iodine (PVP-I) (Sigma-Aldrich, St. Louis, MO, USA, PVP1-100G) and chlorhexidine (CHX) (Sigma-Aldrich, St. Louis, MO, USA, C9394-25ML). Both antiseptics were diluted in warm phosphate-buffered saline solution (PBS) to obtain the working concentrations; three concentrations were used for each antiseptic, as illustrated in Table 2.

### 2.3. 2D Migration Assay—Cell Exclusion Zone Assay

To evaluate cellular migration potential, a cell exclusion zone assay with an Ibidi insert (80209, Ibidi GmbH, Gräfelfing, Germany) was cultured in 24-well plates (CLS3527-100EA, Sigma-Aldrich, St. Louis, MO, USA). For all four cell culture conditions, cells were seeded in the Ibidi insert at a density of 7 × 10^4^ cells in 70 µL of media per reservoir, and allowed to adhere overnight at 37 °C, 5% CO_2_. Following the cell adherence phase, the insert was carefully removed and treated with the two antiseptics in the aforementioned concentrations for 30 s. After treatment, the cells were washed twice with warm PBS solution and replaced with corresponding culture media. Each well was imaged at 0 h, 12 h, and 24 h for cell migration using a Leica C6800 fluorescent microscope with image acquisition system (Leica Microsystems).

### 2.4. 3D Collagen Sprouting Assay

Spheroids for all four cell culture conditions were generated using 96 ultra-low-adherence U bottom plates (650979, Greiner, Bio-One GmbH, Kremsmünster, Austria) and 1% Matrigel (356237, Corning Inc., Corning, NY, USA), as previously described by Ivascu and Kubbies [28]. For each spheroid generation, 2 × 10^3^ cells were used in a working volume of 200 µL of medium/well. Following seeding, spheroid formation and growth were allowed for 72 h in humidified atmosphere conditions at 37 °C, 5% CO_2_, and then treated for 3 min with various antiseptic concentrations. Following treatment, the sprouting assay was performed in pre-coated 24-well plates. The spheroids were dispensed in collagen mix droplets: M199 medium (M0650, Sigma-Aldrich, St. Louis, MO, USA) with 1% Glutamax (35050061, ThermoFisher Scientific, Waltham, MA, USA) and 7.5% sodium bicarbonate (S8761, Sigma-Aldrich, St. Louis, MO, USA) in collagen type I (50201, Ibidi GmbH, Gräfelfing, Germany). After collagen gelation, 500 µL of culture medium was added and the plates were incubated. The spheroids were monitored periodically under phase contrast microscopy, and images were acquired once the sprouting was observed.

### 2.5. Immunofluorescence for α-SMA and Ki67 Markers

Immunofluorescence for α-SMA and Ki67 was performed under all experimental conditions, for both 2D and 3D constructs, 5 days after treatment. Following fixation with 4% paraformaldehyde, primary antibodies for α-SMA (ab32575, Abcam, Cambridge, UK) at 1/500 dilution or 1 µg/mL for Ki67 (ab92742, Abcam, Cambridge, UK) were added for immuno-detection. After being washed in PBS solution, the cells were incubated with the secondary antibody (A32733, ThermoFisher Scientific, Waltham, MA, USA) at 1/500 dilution concentration and Hoechst (H3570, ThermoFisher Scientific, Waltham, MA, USA) nuclear counterstaining at 1/5000 dilution. Following further incubation for 40 min or 1 h, depending on the cell culture system, and additional PBS washes, the samples were stored in PBS at 4 °C for the purpose of image acquisition.

### 2.6. Imaging and Statistical Analysis

Fluorescent microscopy was undertaken with either 5× or 10× magnification on a Leica C6800 fluorescent microscope. For the 3D sprouting assay, confocal imaging was also acquired on a CV7000 confocal microscope. Image analysis was conducted with open-source software package Fiji ImageJ (version 1.51s) [29]. Data collection and analysis were conducted using Excel and GraphPad Prism applications. All data summarized herein were expressed as mean value ± standard error of mean (SEM) and analyzed using one-way ANOVA with Sidak’s multiple comparisons test.

## 3. Results

### 3.1. Cytotoxicity of Antiseptics

When the study was initiated, it became immediately apparent that both top concentrations of CHX and PVP-I (C1 and P1, respectively) mediated a highly cytotoxic effect on all cell constructs, with minimal residual cells left in the culture wells after incubation, as cells detached easily. As a result, it was decided to proceed with the study of the lower concentrations of each antiseptic (C2 and C3, P2, and P3, respectively).

### 3.2. The 2D Cell Exclusion Zone Assay for Migration Assessment

**Figure 1:**
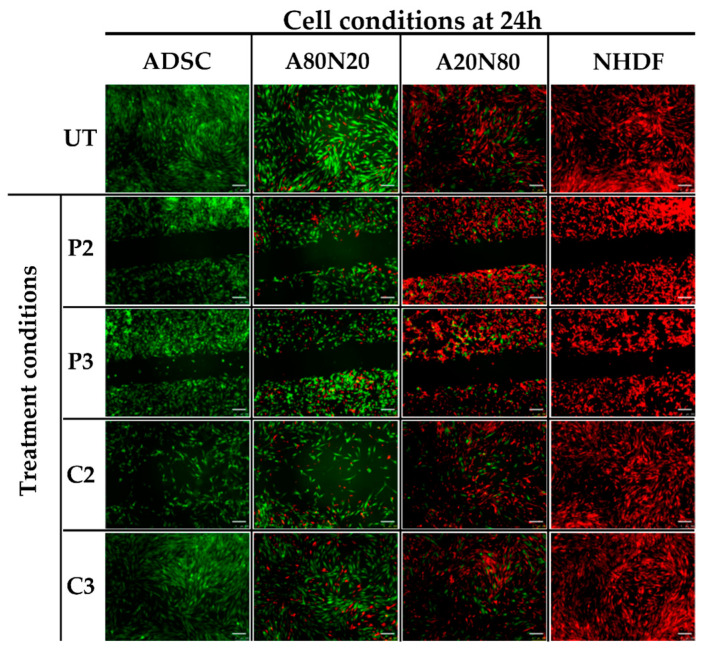
The 2D migration assay. Image acquisition at 24 h following Ibidi insert removal and treatment with gap closed for all cell conditions after treatment with C3, and gap persistence after treatment with both P2 and P3 PVP-I concentrations (UT—untreated; green—ADSCs; red—NHDFs; scale bar—250 µm).

As illustrated in Figure 1, the wound gap under all the untreated (UT) conditions was readily closed as a result of cell migration after 24 h. However, in the cultures treated with PVP-I, the gap was still open after 24 h, with minimal cell migration within the gap regardless of antiseptic concentration, cell type, or coculture ratio. Also, the cells engaged in a single-cell movement pattern, i.e., a mesenchymal-like migration profile, as shown in Figure 1. In contrast, CHX exerted a significantly stronger effect on the migration potential within the stem cell or high stem cell ratio coculture, i.e., A80N20 at high antiseptic concentration, whereas the same concentration of CHX had no significant impact on wound closure in the case of the fibroblast monoculture and the A20N80 construct. At low CHX concentrations (C3), the drug did not exert a significant impact on cell migration, regardless of cell type, and the wound was fully closed 24 h after treatment, as shown in Figure 1.

**Figure 2:**
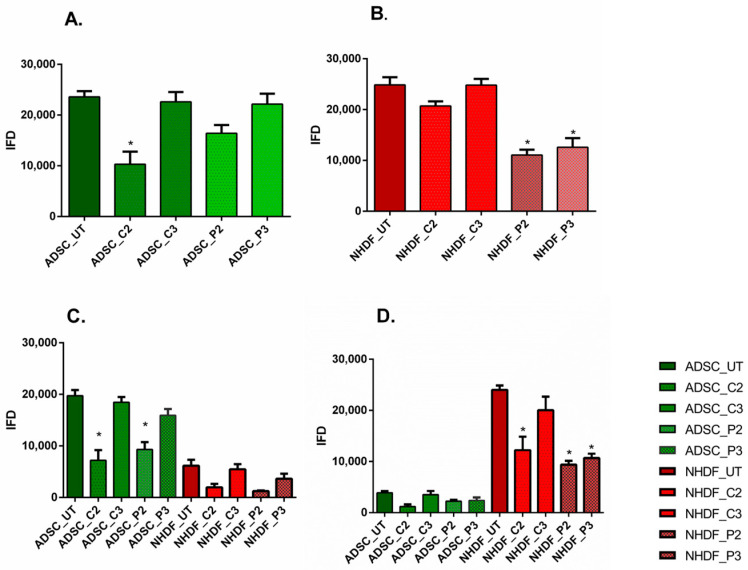
Integrated fluorescent density (IFD) for the 2D migration assay. (**A**) ADSC monocultures, with IFD lower for C2 (* *p* ˂ 0.0001); (**B**) NHDF monocultures with statistical differences for PVP-I concentrations compared with untreated cultures (* *p* ˂ 0.0001); (**C**) A80N20 cocultures, with IFD for stem cell fraction significantly lower for P2 and C2 (* *p* ˂ 0.0001); (**D**) A20N80 coculture, with significant differences IFD for fibroblast fraction (* *p* ˂ 0.0001); all data are displayed as means with standard errors (SEM; UT—untreated).

To evaluate the effect of the tested antiseptics on cell proliferation under different 2D culture conditions, the total integrated fluorescent density (IFD) of each cell type within each assay well was assessed, as illustrated in Figure 2. For ADSC monocultures, the total IFD was significantly lower at C2 compared to the untreated samples (Figure 2A), indicating that modest concentrations of CHX can inhibit stem cell proliferation. Moreover, there was a statistically significant difference between C2 and C3, as well as between C2 and P2. Similarly, for A80N20, the stem cell population was also significantly more affected at higher antiseptic doses of either CHX or PVP-I, in what appears to be a concentration-dependent function (C2 ˂ C3 and P2 ˂ P3), as displayed in Figure 2B (*p* ˂ 0.0001).

The IFD results for the A20N80 culture condition show minor differences between the stem cell populations treated with the two antiseptics in different concentrations. In contrast, for the populations of fibroblasts, significant differences were noted between those untreated and those treated with C2, P2, and P3, where C2 ˂ C3 and P2 ˂ P3 in terms of quantifying the effect of each concentration of antiseptic, as shown in Figure 2C (*p* ˂ 0.0001). For fibroblast monocultures, IFD decreased after treatment, minimally in the case of CHX, and with a statistically significant impact on proliferation at both PVP-I concentrations compared to untreated cultures, as can be seen in Figure 2D (*p* ˂ 0.0001).

### 3.3. The 3D Collagen Sprouting Assay

**Figure 3:**
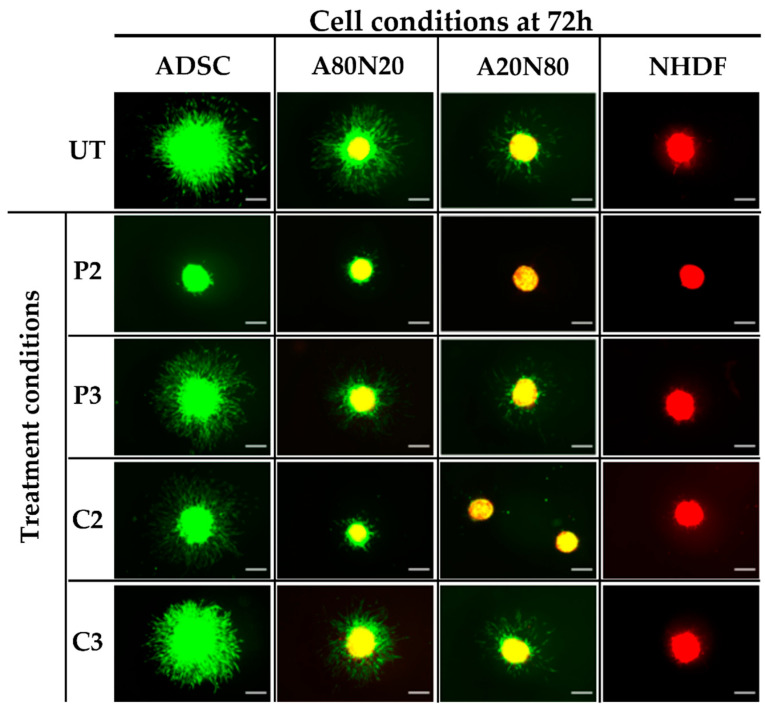
3D sprouting assay. Image acquisition at 72 h following treatment shows reduced sprouting after treatment with P2 and C2 (P2 ˂ C2) and less robust proliferation zone as stem cell ratio decreases (cell conditions with ADSC 100% vs. 80% vs. 20%); (UT—untreated; green—ADSCs; red—NHDFs; yellow shades due to overlay; scale bar—250 µm).

The effects of both antiseptics on cell sprouting in different cultured spheroids were evaluated. For untreated spheroids, the sprouting was more pronounced for ADSC mono- and cocultures, indicating a behavior with a high sprouting tendency of the ADSC cell type. Moreover, in the case of a smaller stem cell ratio, the spikes formed in the outer proliferation zone were less apparent (Figure 3). In contrast, the untreated fibroblast monoculture spheroids exhibited a minimal tendency to sprout, with little or no spike formation, suggesting that ADSCs may be the main cell type driving in the spouting behavior.

**Figure 4:**
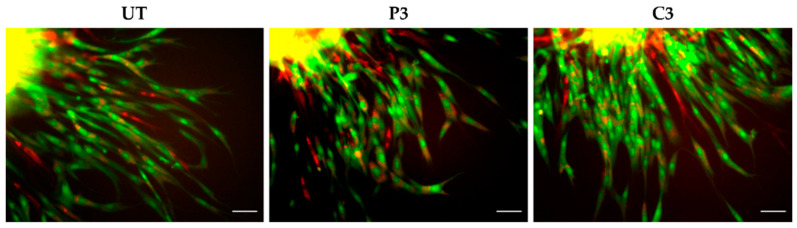
Confocal images representative of A80N20 coculture behavior. After treatment with P3, the spikes contained more fibroblasts compared with the spikes treated with C3, which contained more stem cells (UT—untreated; green—ADSCs; red—NHDFs; yellow shades due to overlay; scale bar—100 µm).

When spheroids were treated with PVP-I and CHX in high concentrations (P2 and C2), all cell conditions displayed a dose-dependent reduction in sprouting features, and this effect was more pronounced for P2 (Figure 3). For untreated A80N20 spheroids as well as for their counterparts treated with C3 and P3, the spikes originated mainly from the stem cell population, as revealed by fluorescent microscopy. When untreated A80N20 spheroids were assessed under confocal microscopy, the sprout features formed from both fibroblasts and stem cells, suggesting a synergized interaction between the two cell types. Despite the strong inhibition of the overall sprouting under treatment with PVP-I (P3), this interaction was maintained. In contrast, for the corresponding spheroids treated with CHX (C3), not only was sprouting greatly inhibited but also fibroblast participation was completely abolished, with the sprouts formed solely by the stem cell population, as shown in Figure 4.

**Figure 5:**
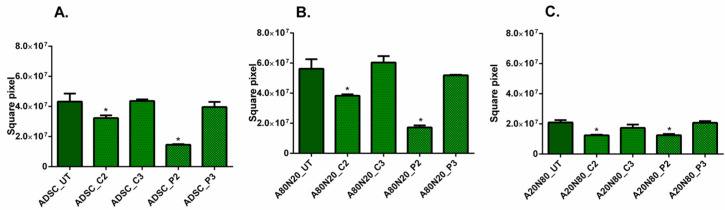
Sprouting area for stem cells in 3D systems with sprouting area reduction after treatment with P2 and C2 (* *p* ˂ 0.0001). (**A**) ADSC monocultures; (**B**) A80N20 coculture; (**C**) A20N80 cocultures; all data are displayed as means with standard errors (SEM; UT—untreated).

Considering the high tendency for stem cells to sprout compared to fibroblasts, the sprouting area was calculated, as shown in Figure 5. For the ADSC monoculture and cocultures, the sprouting area was significantly reduced after treatment with C2 and P2 (*p* ˂ 0.0001). Moreover, the sprouting areas were smaller at lower concentrations of both antiseptics (C2 ˂ C3 and P2 ˂ P3), as can be seen in Figure 5A–C.

### 3.4. α-SMA and Ki67 Markers Evaluation for 2D Constructs

To examine the effects of the studied antiseptics on the α-SMA expression and proliferation status for each cell culture condition, the overall immuno-fluorescence of each marker was normalized to the total Hoechst area, i.e., a surrogate for the total cell number. The results are displayed as ratios to compensate for any change in total cell number due to cell proliferation or cytotoxicity after treatment. Image acquisition was performed 5 days following antiseptic treatment. A minimum of two images were analyzed for each condition.

**Figure 6:**
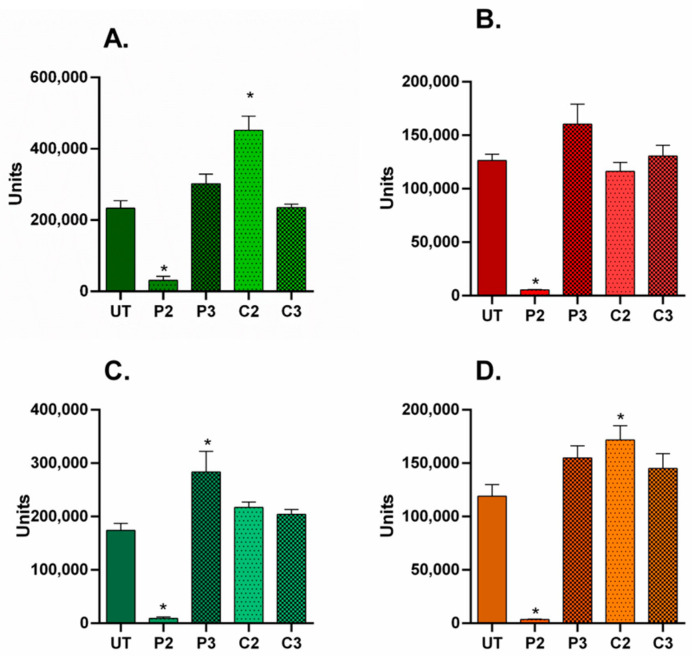
α-SMA/nuclei fluorescent area in 2D culture systems. (**A**). ADSC monocultures—α-SMA/nuclei ratios were significantly reduced following P2 treatment, and they increased under C2 treatment. (**B**) NHDF monocultures—the ratios decreased after P2 treatment, although for P3 and C3 treatments, there were no significant differences between the increased ratios; (**C**) A80N20 cocultures—the ratios significantly decreased for P2 treatment and increased for P3 treatment; (**D**) A20N80 cocultures—the ratios increased significantly following C2 treatment and decreased in the case of P2 treatment (image acquisition was performed 5 days after treatment; for each condition, a minimum of 2 images were analyzed; * *p* ˂ 0.0001); the results represent the overall immuno-fluorescence of α-SMA normalized to the total Hoechst area, i.e., a surrogate for total cell number; all data are displayed as means with standard errors (SEM; UT—untreated).

In all 2D culture conditions, the α-SMA/nuclei fluorescent area ratio values significantly decreased following high-dose P2 treatment, indicating concomitant down-regulation of α-SMA expression across the cell population (Figure 6A–D; (*p* ˂ 0.0001). In contrast, after P3 treatment, the ratios increased for all cell conditions and significantly for A80N20 (*p* ˂ 0.0001). These data suggest that modest PVP-I treatment modulates α-SMA levels under all cell ratios obtained. Moreover, the effects seem to be long lasting, as the α-SMA level variation remained evident 5 days following treatment.

On the other hand, treatment with high-dose CHX (C2) resulted in significantly increased ratio values for ADSC and A20N80, suggesting that α-SMA expression may be dynamically modulated under different cell–cell interaction conditions (Figure 6A–D; *p* ˂ 0.0001).

Regarding the study of proliferative potential, the untreated ADSC monoculture had a significant, high ratio, indicating that the stem cell culture was in a high proliferative state compared to that of fibroblasts. However, the proliferative potential of the stem cell population was evidently suppressed when cocultured with as low as 20% fibroblasts (A80N20), as the Ki67/nuclei fluorescent area ratio was significantly reduced, possibly due to contact inhibition in response to sharing the environment with another cell type.

**Figure 7:**
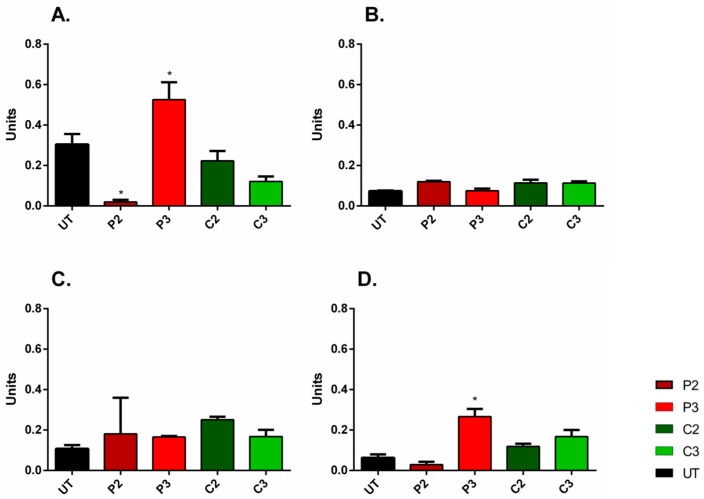
Ki67/nuclei fluorescent area in 2D culture systems. (**A**) ADSC monocultures—the ratio significantly increased following P3 treatment compared to untreated ADSCs and decreased after P2 treatment; (**B**) NHDF monocultures—no significant ratio shifts; (**C**) A80N20 coculture—no significant ratio shifts; (**D**) A20N80 cocultures—the ratios decreased after P2 treatment compared to untreated conditions (image acquisition was performed 5 days after treatment; for each condition, a minimum of 2 images were analyzed; * *p* ˂ 0.0002); the results represent the overall immuno-fluorescence of Ki67 normalized to the total Hoechst area, i.e., a surrogate for total cell number; all data are displayed as means with standard errors (SEM; UT—untreated).

Upon treatment with high-dose P2, the Ki67/Hoechst ratio was significantly reduced for both ADSC monocultures and A20N80 cocultures, suggesting the inhibition of proliferation, whereas the ratio increased significantly after medium P3 treatment of ADSC monocultures, compared to untreated counterparts (*p* ˂ 0.0002). After CHX treatment, although the ratio slightly increased for NHDF monocultures and cocultures and decreased for ADSC monocultures, the different effects were not statistically significant when compared to untreated conditions (Figure 7). In other words, the effects of CHX on proliferation were not as pronounced as those of PVI-I.

### 3.5. α-SMA and Ki67 Markers Evaluation for 3D Spheroid Cultures

Regarding the 3D spheroids, image acquisition was performed 5 days after antiseptic treatment, and a minimum of two spheroids under each condition was evaluated. The results of the semi-quantitative analysis were expressed as α-SMA IFD and spheroidal area ratio values.

**Figure 8:**
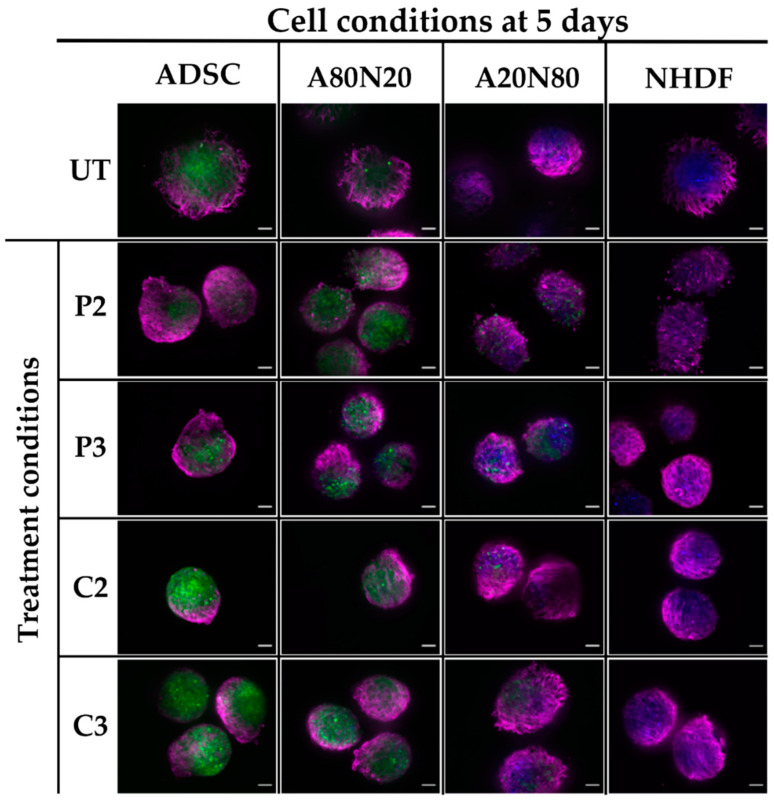
α-SMA qualitative assessment in 3D cultures. α-SMA expression patterns vary with cell type after antiseptic exposure; for ADSC and A80N20 spheroids, α-SMA expression can be observed in a more localized and polarized pattern, especially following CHX, whereas for NHDF and A20N80 α-SMA expression, it appears to spread across the spheroid surface (UT—untreated; green—ADSC; blue—NHDF; magenta—α-SMA; scale bar—100 µm).

For ADSC monotypic and A80N20 spheroids, upon treatment with PVP-I, α-SMA expression induction was strong in a dose-dependent manner, and it appeared to spread across the spheroid surface. In contrast, modest α-SMA expression induction was observed after CHX treatment in a localized manner. For NHDF and A20N80 conditions, α-SMA expression in the spheroid periphery seemed more pronounced after treatment. Following both PVP-I and CHX treatments, α-SMA expression was spread out over the spheroid surface, whereas for ADSC and A80N20, it was more localized and in a polarized pattern (Figure 8).

**Figure 9:**
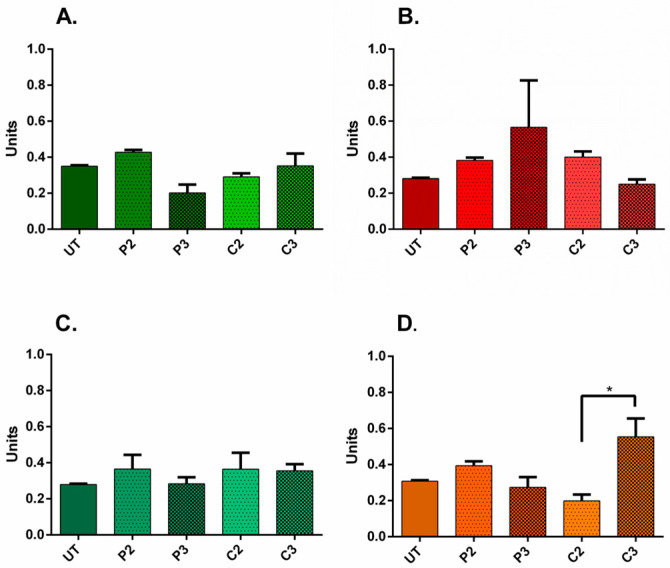
α-SMA/spheroid areas in 3D culture systems. (**A**) ADSC monocultures—the ratio increased especially following treatment with P2. (**B**) NHDF monocultures—the ratio increased following treatment with P3 and decreased slightly after C3 treatment; (**C**) A80N20 cocultures—the ratio increased following treatment, mainly for high concentrations; (**D**) A20N80 cocultures—the ratio increased following C3 treatment, and a statistically significant difference between C2 and C3 treatments was noted (* *p* ˂ 0.037); (image acquisition was performed 5 days after treatment; for each condition, a minimum of 2 spheroids were analyzed); all data are displayed as means with standard errors (SEM; UT—untreated).

Due to variations in spheroid size, apart from the assessment of α-SMA expression patterns following treatment, the ratio between α-SMA IFD and the spheroid area was also evaluated. Changes were observed dependent on antiseptic, concentration, and cell conditions (Figure 9). The only statistically significant difference was between the A20N80 spheroids treated with CHX in the two concentrations, with a ratio increase following C3 (*p* ˂ 0.037). These results suggest that the level of α-SMA varied during the 5 days of one-treatment exposure.

**Figure 10:**
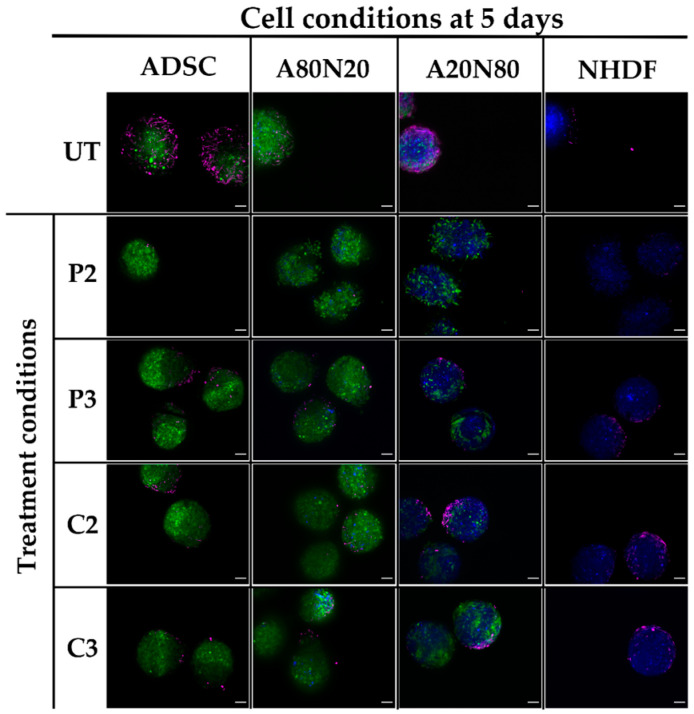
Ki67 qualitative assessment in 3D cultures. Proliferation marker minimally expressed following P2 treatment in all cell constructs; for constructs treated with P3 and CHX, Ki67 expression was observed with a tendency to localize, suggesting a less pronounced effect on proliferation compared with P2 (UT—untreated; green—ADSC; blue—NHDF; magenta—Ki67; scale bar—100 µm).

In the case of 3D cultures, Ki67 could not be quantified, but the expression of this marker seemed greatly inhibited by PVP-I in high concentrations, suggesting a down-regulatory effect of PVP-I on proliferation. For constructs treated with P3 and CHX, Ki67 expression was observed on the spheroid surface, with a tendency to localize in all cell conditions, whereas in untreated conditions, there was spread on the spheroid surface, suggesting a less pronounced effect on proliferation compared to P2 treatment (Figure 10).

## 4. Discussion

Antiseptics are widely used in clinical wound management, especially for controlling wound bioburden [30]. However, some aspects are still controversial in the current state of knowledge and understanding regarding their bactericidal properties, cytotoxicity, and effects on the cell populations that participate in wound healing [4,6,9,31]. The reported data are heterogeneous and based mainly on in vitro studies, most of which have been conducted on fibroblasts and keratinocytes, in 2D culture systems. Such research, restricted to wound-repairing cell types and media, is of limited relevance, and transferability to the clinical practice of wound management is difficult [4,8,9,31]. Similarly, recent in vivo studies have been inconclusive regarding the question of whether various antiseptics have beneficial or negative effects on wound healing. Also, the effects of antiseptics on scarring are insufficiently understood: while one in vivo study on CHX reported a concerning pro-scarring response, other clinical studies have reported beneficial effects on wound healing when using antiseptics for chronic wound management [22,32,33,34].

Thomas et al. demonstrated in vitro that high-dose (0.0032%) CHX treatment decreases the proliferation rate of human fibroblasts, although the opposite occurred when lower concentrations of CHX were used (0.0004%). Moreover, CHX seemed to have a less pronounced impact on migration compared to other antiseptics [3]. Faria et al. evaluated fibroblast morphology following CHX treatment and cell death mechanisms in vitro on mouse fibroblasts. Their study revealed that, at low concentrations, cell death occurred through apoptosis, whereas higher concentrations induced necrosis [14]. However, the authors did not specify the exact composition of the CHX solution, and this limits the transferability of the experimental results to clinical settings. In a different study, Archer et al. reported wound healing delay with impaired fibroblast organization following 0.2% CHX topical application on wounds in a pig model [35].

In our study, the experiments with 0.1% CHX revealed unambiguously that this concentration used in clinical settings had a strong and immediate cytotoxic effect in both 2D and 3D model systems. This outcome may be due to the cells’ direct exposure to the drug or the protein-binding properties of the cellular environment, whereas, in clinical settings, the physiological wound microenvironment is more complex and variable. In 2D culture systems, gap closure was dependent on both concentration and cell type ratio. Treatment with CHX in high concentrations (0.05%) had a less substantial negative impact on cell migration compared to PVP-I, and the effect was more prominent on the stem cell population. At the same time, the gap closure was similar compared to untreated conditions at low concentrations (0.01%). At a higher stem cell ratio of A80N20, treatment with CHX in low concentrations seemed to stimulate the migration of ADSCs within the sprouting spikes. Therefore, our results suggest that small amounts of CHX do not undermine stem cell migration.

The methodological coordinates of the experimental design in such research are crucial for the results to be transferable to clinical settings. Regarding CHX, the current data leave room for debate and further investigation of the effects on wound healing. Abdel-Sayed et al. conducted a survey on the use of CHX in burn management and reported a lack of consensus regarding concentrations, excipient type, and protocols [6]. This could inform in vitro research, such as by accommodating the fact that the concentrations used in clinical settings may not be appropriate in studies where cells would be exposed directly to the antiseptic. As previously mentioned, although 0.1% CHX is usually recommended in clinical settings, for in vitro evaluation, this concentration displayed a high cytotoxic effect on cells directly exposed to it, suggesting that concentrations should be downscaled in correlation with the studied cell exposure.

The α-SMA protein is a well-known marker that has been linked to pathological scarring; its level increases when fibroblasts are activated and adopt a myofibroblast-like phenotype during wound healing [36,37]. Pilloni et al. evaluated the effect of 0.12% CHX on gingival tissue biopsies and reported an increase in α-SMA, proposing that CHX induces a pro-scaring response [22]. In our study, the α-SMA marker was evaluated 5 days after treatment with the two antiseptics using both 2D and 3D cultures. Following CHX treatment, α-SMA expression modulation was observed, with differences between 2D and 3D culture systems. Our results suggest that α-SMA modulation varies with cell type as well as coculture ratio, culture system, and CHX concentration. In 2D cultures, its increase following CHX treatment was modest, suggesting that the studied concentrations of CHX do not have a negative impact on cell proliferation.

As for CHX, the available data on PVP-I are heterogeneous and divergent effects have been reported. Balin et al. evaluated the effects of different concentrations of PVP-I on human skin and fetal lung fibroblasts, reporting proliferation impairment depending on concentration and exposure time, with total growth inhibition at 0.1% and 1% [38]. Liu et al. evaluated in vitro the effects of multiple concentrations of PVP-I on human cell migration, including dermal fibroblasts. In their study, gap closure was inhibited in a wound healing assay at concentrations ≥0.1% after a short exposure time of 3 min [13]. Similarly, our results regarding migration in 2D cultures revealed that gap closure in the wound healing assay was not achieved at 24 h following PVP-I treatment. However, in 3D culture systems, the effect was not as significant for low PVP-I concentrations, with sprouting still ongoing in ADSC monocultures and cocultures after antiseptic treatment. Moreover, for the A80N20 cocultures treated with low doses (0.1%), sprouting spikes were formed from both stem cells and fibroblasts, as displayed in Figure 4, indicating minimal impact on spouting/migration behavior.

Regarding the effects of PVP-I on wound healing, some results of in vitro studies contradict those obtained in vivo. Archer et al. evaluated different antiseptic treatments using a pig wound model and reported that 0.8% PVP-I delayed the healing process [35]. Zhang et al. used a wound model in rats and suggested that 0.05% PVP-I delayed wound healing by increasing the level of inflammatory infiltrate and exerting negative effects on fibroblasts [32]. In contrast, Wang et al. conducted an in vivo experiment on rats and found that 0.5% PVP-I accelerated the healing process by promoting α-SMA and TGFβ expression [33]. According to our results, treatment with PVP-I in a modest concentration of 1% decreased the α-SMA/nuclei fluorescent area ratio in 2D cultures, while at a tenfold lower concentration (0.1%), the ratio actually increased. Although this pattern was observed for all cell conditions, it was significant only for A80N20 conditions. At the same time, at 1% PVP-I, the α-SMA/spheroid ratio increased in the 3D cultures, which mimics physiological conditions better. These results indicating α-SMA modulation following PVP-I treatment in 3D cultures contradict other studies, which invites further and more detailed research before adequate translation to clinical settings, especially as α-SMA is known to be highly expressed in pathological scaring [36]. Moreover, species specificity needs to be considered; take, for instance, an experiment conducted by Wang et al. on a wound healing model using rats, where murine wound healing was mainly mediated by contraction, which is different from the human healing process [39].

Historically, most in vitro studies were performed on skin cell types such as keratinocytes. Stem cells and fibroblasts are the key cell types involved in the wound-healing process. Stem cells are present within the wound microenvironment, either as cells homing within the wound, delivered from an exogenous source, or simply as cell types present within the underlying adipose tissue. Subsequently, this cell type would come in contact with the antiseptics alongside skin cells during wound management, hence the importance of studying the impact of antiseptics on ADSCs [21,26,27,40]. Kim et al. demonstrated in vitro that PVP-I can have negative effects on freshly isolated ADSCs in terms of proliferation, viability, differentiation, and stem cell markers [27]. In our study, when IFD was used to evaluate the effects of the antiseptics, we found that PVP-I had a cytotoxic effect on stem cells similar to that reported by Kim et al. Following treatment with CHX, such a negative effect seems more pronounced on stem cells when CHX is used in high concentrations.

Experimental research into wound healing has generated a wide range of results depending on the studied cell types, coculture ratios, antiseptic concentrations, etc., and awareness is increasing among the medical community about how culture models produce different data. One important recent advance in cell culture techniques was the development of 3D cultures that better mimic the in vivo cell environment. This new biological platform provides the opportunity to develop more physiologically and clinically relevant in vitro models with 3D structures, using appropriate human cells, including genetically manipulated cells, to assess study markers or signals. Although experimental designs with 2D cultures are simple and easy to use, the results may be misleading if transferred to clinical settings with the expectation that outcomes would be similar. In 2D culture models, the cells are organized in a monolayer and are exposed directly to the tested substance, behaving differently than the pharmacodynamics of in vivo microenvironments [41,42]. The noticeable differences in terms of culture behavior across the available research are understandable, given this important distinction between 2D and 3D culture models, as well as the impact of different cell type ratios.

From a clinical point of view, it seems that many approved antiseptics lack a coherent and comprehensive assessment of effects [21]. Even for the substances widely considered key agents in wound management, there is insufficient standardization of testing and evaluation [43]. Moreover, the current standard for antiseptic testing, especially in Europe, DIN-EN-1327, allows for different testing settings and does not define wound conditions in sufficient clarifying detail [31]. Thus, there is a need for developing standardized testing protocols in the context of wound healing management, starting with an experimental design, from the types of antiseptics and concentrations to exposure times and cell models, including cell types, ratios, and culture systems. For example, murine fibroblasts are known to have better tolerance to PVP-I, indicating that cell type matters a great deal in an experimental design featuring this antiseptic [44]. In our study, multiple relevant human cell types were used in different cell ratios in an attempt to mimic various tissue scenarios. The comparison of results emphasizes that cells in a 3D architectural display behave differently towards treatment, which should be considered for future antiseptic testing.

The limitations of the current study are related to the complexity of the image analysis due to spectrum overlaying, i.e., the yellow shades following color channel merging, and the limit of confocal microscopy, especially in 3D spheroid settings. For example, the Ki67 marker was, in particular, difficult to evaluate in a 3D setting due to overlays and its intranuclear position. Thus, the data were assessed semi-quantitatively, using open-source image processing tools with limited features. Due to the number of cell conditions, as well as evaluating them in two culture systems, only two of the most frequently used antiseptics were chosen. Additionally, even if the cells were labeled to enable tracking, for coculture conditions, quantitative assessment for individual cell fractions was difficult. In this regard, we welcome the rapid advances in technology and expect that enhanced quantitation will become feasible in the near future.

## 5. Conclusions

Both studied antiseptics, povidone–iodine (PVP-I) and chlorhexidine (CHX), had a cytotoxic effect on in vitro cultures of adipose-derived stem cells and fibroblasts. This effect varied depending on antiseptic concentrations, cell types, and cell ratios. PVP-I had a more pronounced negative effect than CHX, especially on fibroblasts. Both PVP-I and CHX were found to modulate proliferation and α-SMA expression, implying that further studies are required to assess the full spectrum of effects that exposure to antiseptics in different concentrations can exert on various cocultures of relevant cell populations. An especially pertinent goal is to evaluate long-term effects in terms of wound healing and scarring. Methodologically, the comparative analysis of the results obtained from the 2D and 3D culture systems highlights the phenotypical modulation of cells between 2D and 3D models, suggesting that 3D cultures are more appropriate formats for antiseptic testing in the context of wound healing physiology.

## Figures and Tables

**Table 1:** Cell culture constructs.

Culture Type	Cells	Abbreviation	Cell Ratio
	NHDFs	NHDF	100% NHDF
Monocultures			
	ADSCs	ADSC	100% ADSC
			
		A20N80	20% ADSC with 80% NHDF
Cocultures	ADSCs:NHDFs		
		A80N20	80% ADSC with 20% NHDF

**Table 2:** Experimental concentrations of antiseptics.

Antiseptics	Abbreviation	Concentration	Dilution
	P1	10%	100 mg PVP-I in 1 mL PBS
**PVP-I**	P2	1%	0.5 mL of P1 in 4.5 mL PBS
	P3	0.1%	1 mL of P2 in 9 mL PBS
	C1	0.1%	100 µL of the 20% solution in 19.9 mL PBS
**CHX**	C2	0.05%	2.5 mL of C1 in 2.5 mL PBS
	C3	0.01%	0.5 mL of C1 in 5.5 mL PBS

## Data Availability

The data that support the findings of this study are available from the corresponding author upon reasonable request.

## Abbreviations

α-SMA	α-smooth muscle actin
A20N80	Coculture with 20% adipose-derived stem cells and 80% fibroblasts
A80N20	Coculture with 80% adipose-derived stem cells and 20% fibroblasts
ADSC	Adipose-derived stem cells
CHX	Chlorhexidine
IFD	Integrated fluorescent density
NHDF	Normal human dermal fibroblasts
PBS	Dulbecco’s phosphate-buffered saline
PVP-I	Povidone–iodine
UT	Untreated
